# Mental Illness in the SARS-CoV-2 Pandemic Period: How Does a Collective Stress Factor Affect the Hospitalization Requirement? Data from a Survey of Inpatients Admitted to a Psychiatric University Hospital During the First Year of the SARS-CoV-2 Pandemic

**DOI:** 10.3390/brainsci15060599

**Published:** 2025-06-03

**Authors:** Katharina Marie Steiner, Selin Kilic, Michael Specka, Norbert Scherbaum

**Affiliations:** 1 LVR-University-Hospital Essen, Department of Psychiatry and Psychotherapy, Medical Faculty, University of Duisburg-Essen, 45147 Essen, Germany; selin.kilic@stud.uni-due.de (S.K.); michael.specka@lvr.de (M.S.); norbert.scherbaum@lvr.de (N.S.); 2 Center for Translational Neuro- & Behavioral Sciences (C-TNBS), University Duisburg Essen, 45147 Essen, Germany

**Keywords:** mental illness, SARS-CoV-2 pandemic, psychiatric inpatients, pandemic-related stress, socioeconomic functioning, diathesis–stress model, chronic mental illness

## Abstract

Background/Objectives: According to a diathesis–stress model for the development of mental illness, it is assumed that, in addition to pre-existing individual vulnerability, the occurrence of acute strains is an etiological factor. The SARS-CoV-2 pandemic was a collective massive stressor, which could predispose to a first manifestation of a mental disorder or the exacerbation of a pre-existing mental disorder. The aim of this study was to investigate the effects of the pandemic on the cohort of patients admitted to hospital during the first year of the pandemic. Methods: Patients admitted to inpatient treatment in a university psychiatric hospital in an urban region from April 2020 to March 2021 were interviewed using a systematic questionnaire assessing individual stress factors in the context of the pandemic. On the basis of the interview, clinical practitioners rated the influence of the pandemic on the admission. Results: Six hundred and forty-five patients were interviewed. Only 6.4% showed a strong influence of the pandemic on inpatient admission. This group was characterized by a comparatively high level of socioeconomic functioning. Additionally, the majority of this group had a pre-existing mental disorder. Conclusions: For the majority of patients, the pandemic had only a minor influence on their hospitalization; only for 6.4% was a high impact of the pandemic reported. We hypothesize that this group’s higher socioeconomic functioning in addition to a pre-existing mental disorder made them vulnerable to pandemic-associated limitations. These data confirm a complex diathesis–stress model for the development of mental illness in the context of an acute collective stressor.

## 1. Introduction

Referring to the diathesis–stress model for the etiology of mental disorders, the manifestation of a mental disorder depends on various factors, such as individual vulnerabilities such as a genetic disposition, traumatic experiences, and adverse physical and mental pre-existing conditions [1,2]. In combination with external stressors, including the death of a close relative, separation, conflicts, the loss of a job, or poverty, this complex of factors can lead to the occurrence of a mental disorder if a certain individual level of stress load is exceeded.

The SARS-CoV-2 pandemic was indisputably a significant stressor for mental health: people feared an unknown, possibly life-threatening disease and worried about relatives [3,4]. Drastic changes and restrictions occurred in people’s everyday lives during the pandemic. Many could no longer pursue their professions and were threatened with unemployment [5]. Social life experienced a collapse, as all contact and activities beyond immediate family members or people living in the same household simply ceased, became forbidden and impossible. In addition, living together within close relationships was intensified, which entailed other challenges: homeschooling overwhelmed parents and children, marital conflicts arose and escalated, and the number of cases of domestic violence rose rapidly [6].

But was this extraordinary stress factor significant enough to contribute to the onset of mental illness according to the diathesis–stress model?

The stressors associated with the pandemic resulted in measurable impairments of people’s mental health as an increased level of depressive symptoms, anxiety symptoms, and subjectively experienced stress at the beginning of the pandemic [7,8]. Numerous studies report the effects of the pandemic on mental state in the general population [feelings of stress, anxiety, depressive symptoms [7,9]], and also in specific groups [adolescents, health care workers, the physically chronically ill [10,11,12]]. In the USA, a large survey study examining the prevalence of depressive symptoms in adults before and during the pandemic showed an up to threefold increase in the frequency of depressive symptoms. However, 60–70% of individuals are considered resilient with regard to various stressors [13], which means that they are very likely to get through crises and disasters without significant impairment, since sufficient resources in the sense of a socially supportive environment, self-worth, self-efficacy expectations, and problem-solving orientation are available. In other words, despite a relevant increase in mental symptoms, the majority of people should not be expected to develop a mental illness as a result. An increase in depressive symptoms documented by questionnaires does not equate with a manifestation of a mental disorder such as major depressive disorder [8].

Only few studies consider the impact of the pandemic on the development or exacerbation of mental illness requiring inpatient treatment, especially with regard to the group of chronically mentally ill individuals. Similarly, with regard to other situations associated with exceptional mental stress, there is no clear picture with regard to the question of an increased prevalence of mental disorders. While an increased prevalence of people suffering from depression, anxiety disorders, and PTSD was reported during war times [14], the majority of people seem to survive natural disasters [15]. At this point, the enormous differences between the situations mentioned (war, natural disaster, pandemic) must certainly be emphasized, with the former often meeting the diagnostic criteria of a trauma.

Regarding the impact of the SARS-CoV-2 pandemic on the prevalence of mental disorders, the results are contradictory. In Europe, there seems to have been a slight increase in the prevalence of mental illnesses, especially depression and anxiety disorders, at the beginning of the pandemic, but the prevalence decreased during the further course of the pandemic [16]. With regard to the severity of symptoms of previously existing mental disorders, no clear pattern could be identified in a systematic review in Europe [16]. A study that addressed stress factors of this group of individuals in a qualitative interview showed a high level of distress and widespread difficulties for these individuals in dealing with pandemic-related limitations [17].

Therefore, the question remains of how much impact a collective stress factor such as the SARS-CoV-2 pandemic might have on the occurrence of mental illness in (predisposed) individuals, and/or the worsening of a pre-existing mental disorder. The present study examined how much the circumstances of the daily life of patients admitted to inpatient treatment at a psychiatric hospital had been affected by the public emergency situation of the pandemic, and whether the need for admission to inpatient treatment could be explained by the direct or indirect effects of the pandemic.

## 2. Materials and Methods

### 2.1. Subjects

All adult patients admitted to inpatient and to day clinic treatment at the LVR-University-Hospital Essen, Department of Psychiatry and Psychotherapy and Department of Addictive Behavior and Addiction Medicine were screened for participation during 6 April 2020 and 8 March 2021. The inclusion criterion was adult age at admission. The exclusion criterion was a lack of capacity to understand the study information and to give consent to study participation. This applied to patients with schizophrenia, severe cognitive deficits, e.g., patients with dementia, or to patients with difficulties in understanding the German language.

The study was approved by the ethics committee of the Medical Faculty of the University of Duisburg-Essen (20-9371-BO) and was conducted in accordance with the Declaration of Helsinki [18].

### 2.2. Data Collection

Data were collected via a questionnaire consisting of two parts; the first was completed by the patient and the second by the attending physician or a member of the study team. Since no validated instrument to measure pandemic-associated individual stress factors was available in German at the time of the survey, the study group designed a customized questionnaire on an ad hoc basis. A preliminary version of the questionnaire was used on a pilot sample and checked with regard to comprehensibility and clarity.

The processing time for the questionnaire was approximately 15 min for the patient and 5 min for the physician. The section to be completed by the patient started with sociodemographic information that included age, sex, highest level of schooling, current occupational status, partnership, current living arrangements (people living within the household, presence of garden or balcony), employment status, and current profession. The next section regarded the impact of the pandemic on patients’ lives. Twelve statements included information on the impact of the pandemic on job, short-time work, (increased) care of children or relatives, quarantine, and loss of social and/or private contacts. The participants evaluated these statements in a yes/no format. In addition, there was the answer option “not applicable” (for example, for questions about occupation worked on by patients without occupation). In the next section, nine additional questions assessed the extent of lost support services due to the pandemic (if any) using visual analog scales. This was then followed by 14 questions about restrictions in leisure activities and new activities during the pandemic, which were also recorded using visual analog scales. Nineteen questions recorded subjective psychological stress caused by the pandemic using visual analog scales. The last questions of the questionnaire were yes/no evaluations of the statements “I delayed current admission to a mental health clinic because of the Corona epidemic” and “I sought current treatment at a mental health clinic earlier because of the Corona epidemic”.

The second (third-party assessment) part consisted of four items: admission date, admission diagnosis, and information on the patient’s previous treatment, as well as the summarizing evaluation of the extent to which the need for admission to a psychiatric hospital was related to the SARS-CoV-2 pandemic. The range of the visual analog scale was between the poles “not at all: admission would have been necessary even without the pandemic” and “very strongly: the pandemic is the essential condition for the current mental decompensation”. This assessment included information from the interview with the patient, such as the patient’s own assessment, reported stress factors, whether in connection with the pandemic, the social environment, or the professional situation, and evaluation of the information obtained in relation to the previous medical history.

### 2.3. Statistical Analysis

The clinical raters’ responses regarding the questions on the connection between the pandemic and inpatient admission on the visual analog scale (VAS) were broken down into three groups, with a weak influence corresponding to a rating in the 0–20 mm range, a medium influence corresponding to a rating in the 20.01–79.9 mm range, and a strong influence corresponding to a rating in the 80–100 mm range. These thresholds are based on Heller et al. [19].

Items from the various stress factor domains were combined into indices:

- Work-related changes and strains (change to working from home; working hours reduced; loss of work; loss of orders or contracts; unpaid leave from work);
- Changes and strains in the private environment (care for school children at home; having to care of older relatives/neighbors/friends; having been in quarantine);
- Markedly reduced mental health support options (self-help groups; counselling center; social psychiatric service; outpatient care; help from youth welfare agency; psychiatric outpatient clinic; outpatient therapy; visits to physicians);
- Direct threats from “emotional reaction section” of the interview (being member of a risk group; working in the health sector; severe infection; friends who were infected);
- Markedly reduced daily activities (no more meetings with family or friends; sports, cultural, and church activities were no longer possible).

Frequencies of categorical variables were compared using the chi-square test. The associations between problem indices and “global assessment” of whether admission was associated with the pandemic were calculated using Spearman’s rank correlation coefficient. To further examine the joint association of the five problem indices with “strong influence of the pandemic on hospitalization”, a multiple binary logistic regression analysis was conducted, adjusted for age, gender, and employment status. A model for binary dependent variables was chosen because we were particularly interested in hospitalizations for which only limited doubt existed that they were associated with the pandemic. No pretests or univariate analyses were performed for this research question.

Group differences in means were assessed using Welch-corrected *t*-tests. The results of the statistical tests in the present study were considered significant at *p* < 0.05. The statistical tests were performed using SPSS software (version 17, IBM Company, New York, NY, USA).

## 3. Results

### 3.1. Recruiting Process

**Figure 1:**
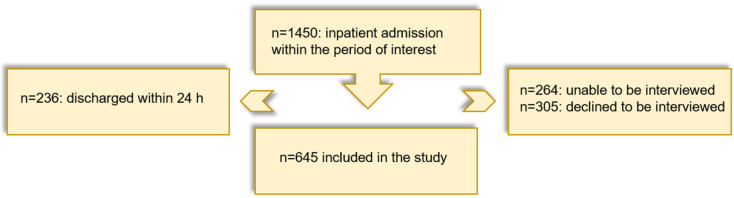
Recruiting process.

In the period from 3 April 2020 to 8 March 2021, 1450 subjects were admitted to inpatient (86.7%) or day clinic (13.3%) treatment at the LVR-University-Hospital Essen. Of these, a total of *n* = 264 turned out to be unable to be interviewed, and another *n* = 305 declined to be interviewed. In addition, *n* = 236 patients were discharged within 24 h of admission, so that no interview could be carried out. A total of *n* = 645 patients were included in the study, and the interview could be completed after the patients’ oral and written informed consent (Figure 1).

### 3.2. Characteristics of the Sample

The mean age of the *n* = 645 patients was 44 (±16.2) years (range: 18 to 92 years). Three hundred and thirty-eight patients were male (52.4%), three hundred and one were female (46.7%), and six were diverse (0.9%). Regarding the main diagnosis (Table 1), the most frequent diagnoses were affective disorders (F3 diagnoses according to ICD-10) in 300 patients (46.5%) and substance-related disorders (F1 diagnoses according to ICD-10) in 251 patients (38.9%).

A markedly different distribution of diagnoses was seen in the 264 patients not eligible for interview, of whom 17 patients were ineligible due to lack of German language skills, 61 patients due to severe cognitive impairment or dementia, and 186 patients due to acute medical conditions. The largest single group of ineligible patients was the group of patients with psychotic disorders (F2 diagnoses in 108 patients, i.e., 40.9%).

The characteristics of the participants with regard to their living and working situation are shown in Table 1. The majority of participants stated that they were not in a partnership and lived alone. In terms of employment status, there was a large group of job seekers and retired people (43.8%), compared to 32.1% employed and 9.5% in training. The vast majority of participants reported having received previous mental health treatment.

### 3.3. Pandemic as a Decisive Factor of the Need for Inpatient Hospitalization?

In the majority of patients (75.2%), treatment providers evaluated that the pandemic had little influence on the need for inpatient admission, while a moderate influence was assumed in 18.2% of cases and a strong influence in only 6.4% of cases.

**Figure 2:**
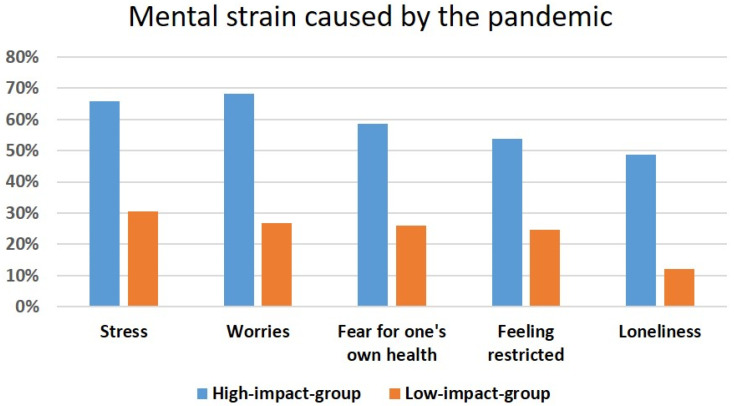
Proportion of patients who rated the given items as “markedly present” in the questionnaire for each of the extreme groups (high-impact vs. low-impact group). Percentages are shown for each group.

In the following, the two extreme groups (high impact of the pandemic on admission to hospital vs. low impact) were compared. In the high-impact group, the patients reported stress, worries, fear for one’s health, feeling restricted, loneliness, and helplessness to a higher extent than patients in the low-impact group. Figure 2 shows the proportion of patients who rated the following items (stress, worries, fear for one’s health, feeling restricted, loneliness, helplessness) as “markedly present” for the group with a high impact of the pandemic compared to the group with a low impact.

**Figure 3:**
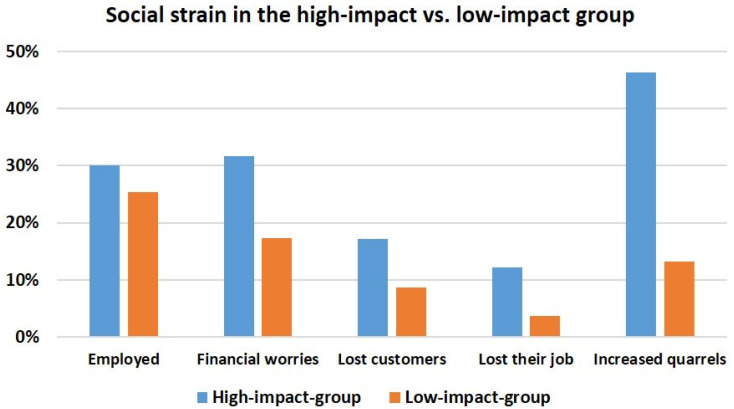
Comparison of the high-impact group with the low-impact group with regard to sociodemographic characteristics and indicated stress factors. Percentages are shown for each category.

Regarding the social burden of the pandemic, the high-impact group was more likely to be employed and reported to a higher extent about financial worries, losing customers, or even their jobs. In addition, increased family quarrels were stated more frequently in this group (Figure 3).

No significant group differences were found with regard to the proportions of the different main diagnoses (for example, the proportion of F3 diagnoses in the high-impact group: 41.5% vs. low-impact group: 46.0%).

Regarding the five indices, i.e., work-related changes and strains (change to working from home; working hours reduced; loss of work; loss of orders or contracts; unpaid leave from work), changes and strains in the private environment (care for school children at home; having to care of older relatives/neighbors/friends; having been in quarantine), markedly reduced mental health support options (self-help groups; counselling center; social psychiatric service; outpatient care; help from youth agency; psychiatric outpatient clinic; outpatient therapy; visits to physicians), direct threats from “emotional reaction section” of the interview (being member of a risk group; working in the health sector; severe infection; friends who were infected), and markedly reduced daily activities (no more meetings with family or friends; sports, cultural, and church activities were no longer possible), the bivariate correlations of the stressor indices with the assessment that there was a strong connection between inpatient admission and the pandemic in the strong-impact group showed statistically significant relationships for work-related strains, daily activities, and direct threats.

Multiple binary logistic regression of the “strong influence of the pandemic on hospitalization” on problem indices was regarded in the next step. The multiple binary logistic regression model included the five indices of pandemic-related problems, in addition to age, gender, and employment as control variables (Table 2). The complete model had a statistically significant relationship with the dependent variable (Chi^2^ value 34.4, df = 8, *p* < 0.001) and showed a small association in the present sample (Nagelkerke’s R-squared 0.11). Adjusting for the covariates and the respective other indices, “Number of work-related strains” and “number of strongly impaired activities”, but not the other three indices had statistically significant associations with “high impact of the COVID pandemic on hospitalization”. For the three non-significant predictors, the means appeared to quite similar between the groups: the mean number of direct threats was 1.2 (SD 1.0) in the high-impact group and 1.1 (1.1) for the remaining patients; for strains in the private environment, the figures were 0.5 (0.7) and 0.3 (0.5), respectively; and for impaired support, they were 0.8 (1.1) versus 0.6 (1.1). The statistically significant predictor “number of work-related strains” had a mean of 0.9 (SD 1.2) in the high-impact group, more than double the mean of 0.4 (0.8) for the remaining patients, and number of strongly impaired activities had means of 6.5 (2.7) and 4.7 (3.2), respectively. Moreover, within the present sample, the *odds* that admission was strongly pandemic-related increased by 59% with every additional work-related strain, and by 14% with each additional impaired activity.

Finally, the impact of the pandemic on the group of patients with no previous psychiatric treatment was evaluated in comparison to the significance of the pandemic in the already pretreated patient group: regarding the proportion of patients with a high impact of the pandemic, there was no statistically significant difference between the groups (*p* = 0.49).

## 4. Discussion

Given the background of the diathesis–stress model, this study investigated whether the SARS-CoV-2 pandemic had a major influence among various factors that could have an impact on the occurrence or exacerbation of a mental disorder of such a severity that inpatient or day clinic treatment in a psychiatric hospital was needed. The cohort studied was on average around 44 years old and consisted of roughly equal numbers of men and women; most of the participants had been admitted to full or partial inpatient treatment for affective disorders or substance-related disorders. It was hardly possible to make statements about the group of acutely psychotic or cognitively impaired patients on the basis of the available data.

According to the treating professionals, a strong influence of the pandemic was assumed only in a small minority of patients. Conversely, this means that the majority of inpatient admissions during the first year of the pandemic were not primarily due to the stressors and burden related to the pandemic, which can be explained by several factors: It is well known that the majority of psychiatric inpatients suffer from chronic relapsing disorders with multiple disadvantages in their social lives [20]. Therefore, there is a high proportion of unemployed and (early) retired in this group. A notable proportion lives alone (49.1%; in comparison, only 20.3% of the general population in Germany lived alone at that time [21]) or in residential facilities and participates in social life only to a limited extent. This was also reflected by our data showing pretreatment in 90.5% of all cases. Only 32.1% reported being professionally active; 25.7% were job seekers (in contrast to the general unemployment rate in Germany in 2020 of 6.3%). Many pandemic-associated stresses thus did not materialize in this group: those who live alone do not suffer from marital strife and homeschooling. Those who are looking for work anyway do not suffer from a poor business situation or short-time work. However, the chronic stress factors in this group (loneliness, limited social participation) did not increase significantly during the pandemic.

The small minority of patients with a high impact of the pandemic on their admission reported above-average stress, worries, fear for their own health, feeling restricted, loneliness, and helplessness, which reflects the typical psychological stress associated with the pandemic [7,8]. In terms of sociodemographic characteristics, this group was more likely to be professionals. Their work situation seemed to have been particularly affected by the pandemic, as reflected by the higher proportions of those who reported loss of assignments, loss of jobs, and financial worries. This was statistically confirmed by the multiple binary regression analysis; the number of work-related stressors and the number of severely impaired activities were significantly associated with the assessment of a strong influence of the pandemic on inpatient admission. According to this analysis the high-impact group is characterized by a higher level of socioeconomic functioning, which could have made them vulnerable to the respective pandemic-associated limitations. Only those who were previously employed could suffer from losing their job; only those who are in close contact with other people suffer when disputes and conflicts arise. And only those who usually engage in many cultural, social, or sporting activities will unfortunately suffer if these activities are discontinued.

Interestingly, the high-impact group was not overrepresented within the patient group without pretreatment, or vice versa: it was not primarily first-time help-seekers for whom a high impact of the pandemic was reported. This signifies that in addition to vulnerability to the psychosocial stressors of the pandemic, a pre-existing mental illness (which already made it necessary to access the healthcare system) appeared to be relevant for the current inpatient admission. The group without pretreatment showed some similarities with the high-impact group, such as a comparatively higher proportion of working patients and, correspondingly, a higher proportion of work-related strains than in the whole cohort. It is plausible to assume that those seeking psychiatric help for the first time are generally better socially integrated than people who have been suffering from a chronic mental illness for many years. However, the proportion of patients without pretreatment in this cohort was relatively small (9.5%). In order to analyze whether there was a change in our clinical sample regarding the proportion of patients with previous inpatient treatment in our hospital before versus during the first year of the pandemic, data from the medical control system at the time of the survey were compared with a historical sample. In the period from 2020/04/01 to 2021/03/31, 45% of patients had already been admitted to inpatient or day clinic treatment at the LVR-University-Hospital Essen in their history. For a historical sample, the respective figure in the period from 2018/04/01 to 2019/03/31 was very similar (44.9%).

### Limitations

Several limitations of the present study should be named. Firstly, the present study is not suitable to answer the questions causally. On the basis of the available data, it is only possible to take a descriptive look at the information provided by a large number of psychiatric inpatients during this period. Secondly, the heterogeneity of the sample with regard to mental disorders complicates the interpretation of the data. Thirdly, the questionnaire used is an instrument that has not yet been formally validated and records the subjective assessment of patients and practitioners. The assessment of the connection between the pandemic and inpatient admission by the treating therapist depends on numerous factors, whereby systematic errors cannot be ruled out. Fourthly, the number of patients who could not be included due to lack of capacity to consent must be taken into account at this point. In this cohort, F0 and F2 diagnosis groups were clearly overrepresented, thus, of patients included, namely those with affective disorders and substance-related disorders. On the other hand, a similar picture would be expected within the group of non-included patients with regard to chronicity and socioeconomic characteristics, as in the analyzed cohort. Lastly, despite the high number of patients included in the study, the number of patients of special interest, namely those with assumed influence of the pandemic (*n* = 41; 6.4%), turned out to be relatively small, which limits the observations of this group.

## 5. Conclusions

The main finding of the present study is that during the first year of the SARS-CoV-2 pandemic only a minor correlation was observed between the admission to hospital treatment and the stress due to the pandemic. The group with a strong influence of the pandemic was characterized by comparatively high levels of socioeconomic functioning, which made them more vulnerable to restrictions related to the pandemic than socially disintegrated patients. In addition, with a pre-existing mental illness (as reflected by reported pretreatment), the pandemic was an additional risk factor in patients with an assumed strong influence of the related stressors. In contrast, first admissions without previous treatment in the health care system for mental disorders were only observed in a small minority (9.5%). This might be a further indication of the resilience of the majority of the population. An increase in the burden with some symptoms during the pandemic must not be confused with an (especially the first) occurrence of a mental disorder of a severity requiring inpatient treatment. Only in a small number of cases were previously mentally healthy people first admitted to inpatient treatment during the pandemic. In summary, this study emphasizes the multidimensional approach of a diathesis–stress model for the development and exacerbation of mental disorders with corresponding implications for the content of psychotherapy. The pandemic only appeared to be the decisive factor for the exacerbation of a mental illness if there was a corresponding interplay of vulnerability (mostly an already known mental disorder before the pandemic) and several serious stress factors related to the pandemic. In addition, the social disadvantage of people with chronic mental disorders let them be (paradoxically and in some way cynically) less at risk of being exposed to the psychosocial stressors of the pandemic.

## Figures and Tables

**Table 1:** Sociodemographic, diagnoses, and pretreatment in the sample.

Sociodemographic, Diagnoses, and Pretreatment in the Sample				
	Proportion [%]			Proportion [%]
Gender			Partnership	
Male	52.4		No partner	56.3
Female	46.7		Living apart	14.1
			Cohabitating	29.3
			Not specified	0.3
Highest school qualification			Living together with others	
SSD 9 years	25.7		Living alone	49.1
SSD 10 years	28.7		Living with partner	19.7
High school diploma	26.5		Living with family	13.2
University degree	11.9		Living with other people	17.4
No school diploma	7		Not specified	0.6
Employment			Previous mental health treatment	
Working	32.1		No treatment at all	9.5
In training	9.5		Outpatient pretreatment	22
Retirees	18.1		Inpatient pretreatment	7.6
Job seekers	25.7		Out- and inpatient pretreatment	59.8
Other	14.6			
Main diagnosis [ICD-10]				
F0	0.8			
F1	38.9			
F2	6.5			
F3	46.5			

The percentages of patients with regard to their life and work situation are presented. SSD 9 years: secondary school diploma after 9 years, SSD 10 years: secondary school diploma after 10 years.

**Table 2:** Coefficients of the multiple binary logistic regression model.

	B	s.e.	Wald Statistic	df	*p*	Odds Ratio (95% CI)
Number of direct threats	−0.04	0.12	0.09	1	0.76	0.96 (0.76–1.22)
Number of changes and strains in the private environment	0.09	0.23	0.14	1	0.71	1.09 (0.7–1.72)
Number of work-related strains	0.47	0.15	9.55	1	0.002 *	1.59 (1.19–2.15)
Number of strongly impaired activities	0.13	0.04	9.51	1	0.002 *	1.14 (1.05–1.23)
Number of impaired mental health/medical support	0.01	0.12	0.00	1	0.95	1.01 (0.8–1.28)
Age	0.01	0.01	0.37	1	0.54	1.01 (0.99–1.03)
Employed	−0.07	0.34	0.05	1	0.83	0.93 (0.48–1.82)
Male gender	−0.36	0.27	1.79	1	0.18	0.70 (0.41–1.18)
Constant	−3.18	0.58	30.54	1	0.00	0.04 (0.01–0.13)

Coefficients of the multiple binary logistic regression model are displayed. Age, employment status, and gender were used as covariates. B: regression coefficient, s.e.: standard error, df: degrees of freedom, CI: confidence interval. Significant results are indicated by bold print and asterisks.

## Data Availability

The data are made available by the authors on reasonable request.
